# Low-level Laser Therapy to the Mouse Femur Enhances the Fungicidal Response of Neutrophils against *Paracoccidioides brasiliensis*

**DOI:** 10.1371/journal.pntd.0003541

**Published:** 2015-02-12

**Authors:** Eva Burger, Ana Carolina S. C. Mendes, Giulia M. A. C. Bani, Maísa R. P. L. Brigagão, Gérsika B. Santos, Luiz Cosme C. Malaquias, Jorge Kleber Chavasco, Liana M. Verinaud, Zoilo P. de Camargo, Michael R. Hamblin, Felipe F. Sperandio

**Affiliations:** 1 Department of Microbiology and Immunology, Institute of Biomedical Sciences, Federal University of Alfenas (UNIFAL-MG), Alfenas, Minas Gerais, Brazil; 2 Department of Biochemistry, Institute of Biomedical Sciences, Federal University of Alfenas (UNIFAL-MG), Alfenas, Minas Gerais, Brazil; 3 Department of Structural and Functional Biology, Institute of Biology, State University of Campinas (UNICAMP), Campinas, São Paulo, Brazil; 4 Department of Microbiology, Immunology and Parasitology, Federal University of São Paulo (UNIFESP), São Paulo, São Paulo, Brazil; 5 Wellman Center for Photomedicine, Massachusetts General Hospital, Boston, Massachusetts, United States of America; 6 Department of Dermatology, Harvard Medical School, Boston, Massachusetts, United States of America; 7 Harvard—MIT Division of Health Sciences and Technology, Cambridge, Massachusetts, United States of America; 8 Department of Pathology and Parasitology, Institute of Biomedical Sciences, Federal University of Alfenas (UNIFAL-MG), Alfenas, Minas Gerais, Brazil; University of California San Diego School of Medicine, UNITED STATES

## Abstract

Neutrophils (PMN) play a central role in host defense against the neglected fungal infection paracoccidioidomycosis (PCM), which is caused by the dimorphic fungus *Paracoccidioides brasiliensis* (*Pb*). PCM is of major importance, especially in Latin America, and its treatment relies on the use of antifungal drugs. However, the course of treatment is lengthy, leading to side effects and even development of fungal resistance. The goal of the study was to use low-level laser therapy (LLLT) to stimulate PMN to fight *Pb in vivo*. Swiss mice with subcutaneous air pouches were inoculated with a virulent strain of *Pb* or fungal cell wall components (Zymosan), and then received LLLT (780 nm; 50 mW; 12.5 J/cm2; 30 seconds per point, giving a total energy of 0.5 J per point) on alternate days at two points on each hind leg. The aim was to reach the bone marrow in the femur with light. Non-irradiated animals were used as controls. The number and viability of the PMN that migrated to the inoculation site was assessed, as well as their ability to synthesize proteins, produce reactive oxygen species (ROS) and their fungicidal activity. The highly pure PMN populations obtained after 10 days of infection were also subsequently cultured in the presence of *Pb* for trials of protein production, evaluation of mitochondrial activity, ROS production and quantification of viable fungi growth. PMN from mice that received LLLT were more active metabolically, had higher fungicidal activity against *Pb in vivo* and also *in vitro*. The kinetics of neutrophil protein production also correlated with a more activated state. LLLT may be a safe and non-invasive approach to deal with PCM infection.

## Author Summary

PCM triggers a typical granulomatous inflammatory reaction with PMN playing a major role; these inflammatory cells are crucial in the initial stages of PCM, participating in the innate immune reaction and also directing the acquired immune response in the later stages. In some PCM patients, these immune mechanisms are insufficient to eradicate the infection, and need to be boosted with antifungal drugs that have to be administered for long periods and can show serious side-effects. We aimed to develop a novel and safe way to activate PMN through low-level laser irradiation of the bone marrow in the mouse femoral medulla. LLLT increased PMN viability and activation, shown by a significantly greater production of protein and ROS, as well as a higher fungicidal capacity; PMN even retained their higher metabolic activity and fungicidal ability after a second exposure to the pathogenic fungus *in vitro*. This is the first time that LLLT has been shown to increase the immune response against a fungal infection, and could be a promising and safe technique to be used with antifungal drugs in PCM.

## Introduction

*Paracoccidioides brasiliensis* (*Pb*) is a non-sexual thermodimorphic fungus that exists in either a mycelium or a yeast form; the latter being pathogenic to humans and can cause an important and neglected systemic infection called paracoccidioidomycosis (PCM). The likelihood of infection and its severity depends on the amount of inhaled fungi as well as the immunological status of the individual [1]. Patients with immune suppression or defects in immune cell activation are more susceptible to PCM [2,3].

PCM presents as a primary acute infection that is later transformed to a chronic phase. However, regardless of the stage of the disease, inflammatory cells play a central role in fighting *Pb*, particularly the neutrophils or polymorphonuclear cells (PMN) [4]. Besides the production of several direct antimicrobial factors, PMN may also secrete cytokines, chemokines and growth factors [5] that promote the host response against the infection. PMN are not only critical for the innate immune response, but can also help the adaptive immune response by interacting with B lymphocytes [6], T cells [7] and dendritic cells [8].

Previous studies have reported prominent neutrophilic infiltrates in paracoccidioidomycotic lesions in experimental animal models such as hamsters [9,10], rats [11] and also in tissue samples from patients [12]. Along with macrophages and plasmocytes, PMN are conspicuous in PCM granulomatous lesions and lead to altered morphology of the nearby fungal cells [13]. The immunological defense against fungi relies on the interaction between specific components of the fungal cell (pattern-associated molecular patterns or PAMPs) and pattern recognition receptors (PRRs) on host phagocytes. Through the binding of PAMPs to PRRs, a signaling cascade is initiated leading to release of pro- and anti-inflammatory cytokines linked to phagocytosis and intracellular fungal cell killing [14].

PMN can also help to eradicate pathogens via phagocytosis and the generation of reactive oxygen species (ROS) during the respiratory burst [15]. Nevertheless, despite the crucial role of inflammatory cells, they are usually not sufficient to entirely eliminate the *Pb* on their own, and patients usually need additional antifungal drug therapy [16]. Itraconazole, for instance, is effective in treatment of PCM, although its use may allow the relapse of the disease several months after discontinuation of the drug therapy [17].

Antifungal medication can also lead to diverse side-effects including dizziness, headaches, epigastric pain [16] and, more importantly, to the development of drug-resistance in the targeted microorganisms [18]. Therefore we asked whether there could be a novel way of activating PMN through the safe and non-invasive technique of low-level laser therapy (LLLT). LLLT uses non-thermal and non-ionizing light irradiation that has been successfully used for acceleration of healing as well as reduction of pain and inflammation [19–21].

Although LLLT may often work as an anti-inflammatory modality [22], it can, depending on the parameters, also trigger the activation of immune cells [23,24] and the activation of pro-inflammatory pathways [25]. While the activation of PMN by LLLT is not a completely novel process and has been reported *in vitro* [26,27], the use of LLLT to help the organism to combat PCM is a new idea; thus, we aimed to assess the fungicidal capacity of PMN after LLLT by characterizing these cells on secretory protein levels, mitochondrial activity and ROS discharge following a first and second exposure to *Pb*.

## Materials and Methods

### Ethics statement

This research was carried out in accordance with the ethical principles required for animal experimentation and was approved by the Ethics Committee on Animal Research of the Federal University of Alfenas, under the protocol registration No. 477/2012. The animal procedures were conducted in accordance with the guidelines with animal care and use committee at Brazil`s National Council for the Control of Animal Experimentation.

### Animals

Swiss outbred female mice were kept in controlled temperature rooms and fed with sterile food and distilled water *ad libitum*. The animals were kept under a 12 light/12 dark cycle, and it was ensured that personnel did not enter the mouse facilities during the dark cycle.

### Preparation of fungal cells and fungal cell wall components

Isolates of the highly virulent *Paracococcidioides brasiliensis Pb*18 strain [28] were grown in semi-solid culture of Fava Netto [29], with the culture medium replaced routinely every 7 days. A polysaccharide preparation known as Zymosan, derived from cell walls of the yeast *Saccharomyces cerevisiae* and containing β-D-glucan was commercially obtained (Sigma-Aldrich, St. Louis, MO, USA).

*Pb*18 cells or Zymosan were washed with sterile 0.9% saline solution and centrifuged (5810R Centrifuge, Eppendorf, NY, USA) 3X at 1300*g*. A fungal suspension containing 5x10^7^ yeast cells/ml was measured using a cell viability count after staining by the vital dye Janus Green B [30] and a hemocytometer. Zymosan particles were directly counted by hemocytometer.

### Infection and inoculation of mice

At 6 weeks of age and weighing approximately 25g, the animals received an “air pouch” as described by Harmsen and Havell in 1990 [31] and modified by Meloni-Bruneri et al. in 1996 [32]. An air pouch was produced in the dorsal region of mice by a subcutaneous injection of 2 ml of air; then, 0.1 ml of either the fungal suspension, Zymosan or saline was subsequently injected in the same region. It was previously shown by our group that *P*. *brasiliensis* elicits a marked neutrophil recruitment *in vivo* after air-pouch inoculation of the virulent *Pb*18 in mice; the mechanism behind this cell recruitment is probably due to chemotactic factors produced by the fungi and injured tissue [32]. In order to show that the PMN recruitment was truly invoked by the fungal cells or its derivatives and not by the air-pouch procedure itself, two additional groups were created and consisted of saline solution inoculation either followed or not by LLLT.

### Experimental groups and laser irradiation

The animals were divided into four groups, namely, group 1: animals infected with *Pb*18 and light irradiated; group 2: animals infected with *Pb*18 but not irradiated; group 3: animals inoculated with Zymosan and light irradiated; and group 4: animals inoculated with Zymosan and not irradiated.

LLLT was performed on two points on each hind leg; the laser device used was a Twin flex laser (MMO, São Carlos, SP—Brazil) with a spot size of 0.04 cm^2^. The laser parameters were: continuous wave near-infrared light (780nm) to deliver 12.5 J/cm^2^ with a 50 mW total power; the total energy was 0.5 J per point (30 seconds per point). Our goal was to reach the bone marrow of the femoral bones, where the process of blood cell formation, known as hematopoiesis, including neutrophils is originated [33]. LLLT was performed on alternate days, with the animals first irradiated immediately after infection and last just before the neutrophil collection. In that way, the animals were irradiated on day 0 (infection or inoculation); day 2; day 4; day 6; day 8; and day 10 (collection of PMN); thus, 6 irradiations were performed.

### PMN isolation

PMN were collected 10 days after the infection or the inoculation of the mice. The animals were anesthetized with a lethal dose (0.5 ml of a 10% ketamine hydrochloride and 2% Xylazine solution); after a skin flap procedure was performed, the cells were collected and placed in sterile tubes with the help of a sterile glass Pasteur pipette and were subsequently dissociated by pipetting. The cells were then transferred and stored in Falcon tubes containing RPMI (Sigma-Aldrich, St. Louis, MO, USA) with 10% Fetal Bovine Serum (FBS—Sigma) and were kept refrigerated (2–6°C) to be used for the subsequent experiments described below. The cells were quantified using a hemocytometer and the cell viability was assessed with 0.2% Trypan blue (Sigma).

For the fungal co-culture experiment with PMN, the refrigerated cells were centrifuged at 1780*g* and washed once before suspension in 15 ml of RPMI; then, the cells were quantified in a hemocytometer and viability was assessed with Trypan blue. The final concentration was adjusted to 10^6^ PMN/ml. *Pb* cells were 3X washed with sterile 0.9% saline and centrifuged at 1300*g* and re-suspended in RPMI with 10% FBS. The concentration of the suspensions was adjusted according to the concentration of the obtained phagocytic cells, so that the cultures remained in a proportion of 1 *Pb* to 25 PMN to be further utilized for the evaluation of PMN metabolic activity, ROS quantification and quantification of viable fungi. Cells were counted in an hemocytometer and the *Pb* viability was determined by the staining with Janus Green B vital dye [30].

After adjusting the PMN suspension (10^6^ PMN/ml), and the *Pb* fungal suspension (4x10^4^ cells/ml) to provide the co-cultivation mixture (1ml of each suspension), which was added to 12 well plates (Corning, New York, USA), the plates were incubated at 5% CO_2_ and 37°C for 2, 6 and 18 hours. After incubation, the cells were centrifuged at 1780*g* and the PMN pellets had their viability assessed by 0.2% Trypan Blue staining.

### Assessment of mitochondrial activity

In a 96 well plate (Corning) we added 100 μl of a 10^6^ *Pb*18/ml suspension and 100 μl of a 5x10^6^ PMN/ml suspension maintaining a ratio of 1:5 (*Pb*:*PMN*). The experiment was performed in triplicate. After 2 hours of incubation (5% CO_2_ and 37°C) we added 20 μl of MTT (Sigma) to the wells. The plate was further incubated for 4 hours. The supernatant was removed, leaving only the pellet at the bottom of each well. Then, 200μL of DMSO (Sigma) was added to each well and the plate was read in a microplate reader at 540nm (Anthos Zenyth 200, Biochrom, Cambridge, UK).

### Evaluation of the metabolic activity of PMN

The BCA method (Sigma) allows colorimetric detection and quantification of the total level of protein in a solution. This method combines the reduction of Cu^2+^ to Cu^+^ by protein in an alkaline medium (the Biuret reaction) with highly sensitive and selective colorimetric detection of the Cu^+^ ion using a reagent containing bicinchoninic acid [34]. The assays were performed in triplicate and the optical densities were measured in a microplate reader (Biochrom) at a wavelength of 560 nm. The results were expressed in mg of protein/ml, comparing the optical density with a standard curve containing known concentrations of bovine serum albumin (BSA—Sigma). The calibration curve was made with a BSA solution of 10 μg/ml at 6 different protein concentrations: 10; 5; 2.5; 1.25; 0.67 and 0.33 μg/ml. The total protein concentration of each sample was calculated by pipetting 50μl of previously disrupted cells (ultrasonic method) along with 200μl of BCA. All samples were pipetted in triplicate and the results corresponded to the mean of the values obtained after blank (RPMI medium) subtraction for PMN cultured *in vitro* or co-cultivated with *Pb*18.

### Quantification of reactive oxygen species

The quantification of reactive oxygen species produced by the PMN oxidative “burst” was carried out by the luminol chemiluminescence assay. PMN were obtained from the experimental groups and adjusted to a suspension of 1x10^6^ PMN cells/ml; for the co-cultivation experiments PMN cells were adjusted to the proportion of 1 *Pb* to 25 PMN (*Pb* concentration 4x10^4^ cells/ml, PMN 1x10^6^ cells/ml). Luminol (Sigma) was used as the substrate for this assay; 135 μl of the PMN suspension was added into a cuvette along with 30 μl of luminol; followed, for the co-cultivation experimental groups, by 135μl of the *Pb*18 suspension. A luminometer (Glomax 20/20 Luminometer, Promega, USA) was used to measure the chemiluminescence signal over 30 minutes. Positive (PMA—phorbol myristate acetate, Santa-Cruz, Brazil) and negative (DPI—diphenyleneiodonium, Sigma) controls were employed.

### Quantification of viable *Pb* through colony forming units

The material collected from the subcutaneous air-pouches was immediately centrifuged at 1780*g* (5810R Centrifuge, Eppendorf, NY, USA). The pellets were re-suspended in 100μl PBS, and spread on Petri dishes with the aid of a sterile Drigalski spreader. Similarly, after centrifugation at 1780*g*, 100μl of PMN/*Pb* mixed suspensions obtained after 2 hours of co-cultivation were spread on Petri dishes. The experiments were performed in triplicate. The fungal growth on plates was allowed to take place over a period of 12 days, when a paintbrush marker was used to highlight the colonies. The culture medium used in this procedure was BHI agar (HiMedia Laboratories, India) supplemented with 1% glucose, 30% growth factor mixture produced by the fungus itself and 10% FBS, as described by Singer-Vermes et al. in 1992 [35].

### Statistical analysis

The results were analyzed using the Shapiro-Wilk normality test and were all considered to have a normal distribution. Groups were compared using a Student`s T test with the level of significance set at 5%. The software used for the analyses was Graph-Pad Prism 6 (GraphPad Software, Inc; La Jolla, CA 92037, USA).

## Results

### PMN recruitment and viability

**Fig 1:**
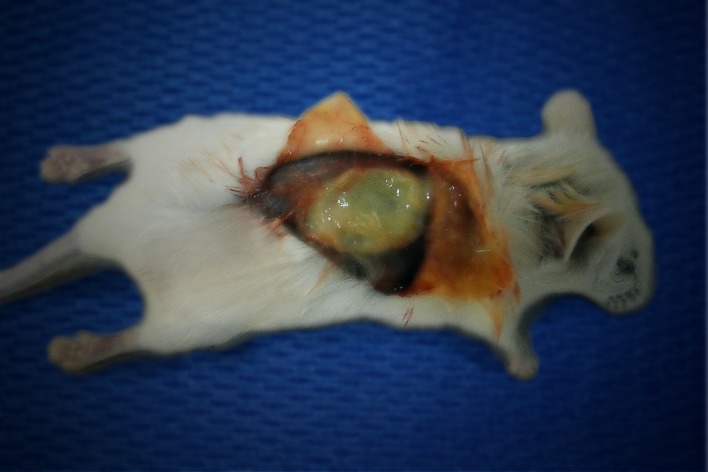
Subcutaneous air-pouch. Clinical appearance of the air-pouch on the dorsum of the mouse after skin flap procedure.

**Fig 2:**
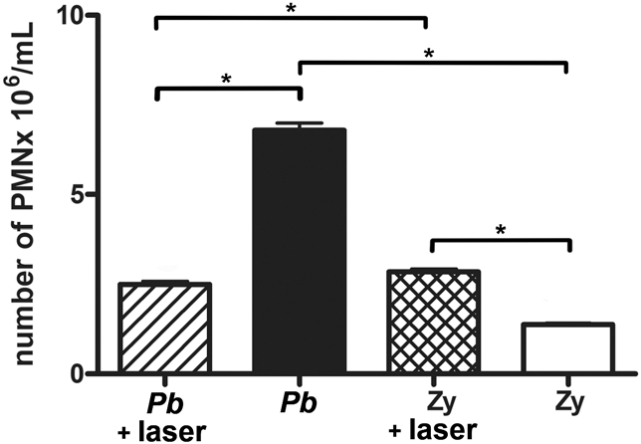
Extraction of PMN from the air-pouch. Absolute number of PMN at extraction time for both irradiated and non-irradiated mice of *Pb* and Zymosan (Zy) groups; p = 0.0001 (*) between non-irradiated and irradiated PMN of *Pb* groups; p = 0.0001 (*) between non-irradiated and irradiated PMN of Zy groups; p = 0.0001 (*) between PMN from non-irradiated *Pb* and Zy groups; p = 0.0399 (*) between PMN from irradiated *Pb* and Zy groups.

The animals inoculated with saline showed no neutrophils at the site of infection even after 10 days (S1 Fig.), which clearly showed that neither the air-pouch procedure alone nor the laser irradiation alone was responsible for the PMN recruitment. The PMN produced by the inflammatory stimuli (either *Pb*18 infection or Zymosan inoculation) were harvested from the subcutaneous air-pouches (Fig. 1), and whilst the total number of PMN recruited to these air pouches was significantly diminished (p = 0.0001) when LLLT was used after the *Pb* infection, the number of PMN was significantly increased (p = 0.0001) when LLLT was used after mice were inoculated with Zymosan (Fig. 2).

**Fig 3:**
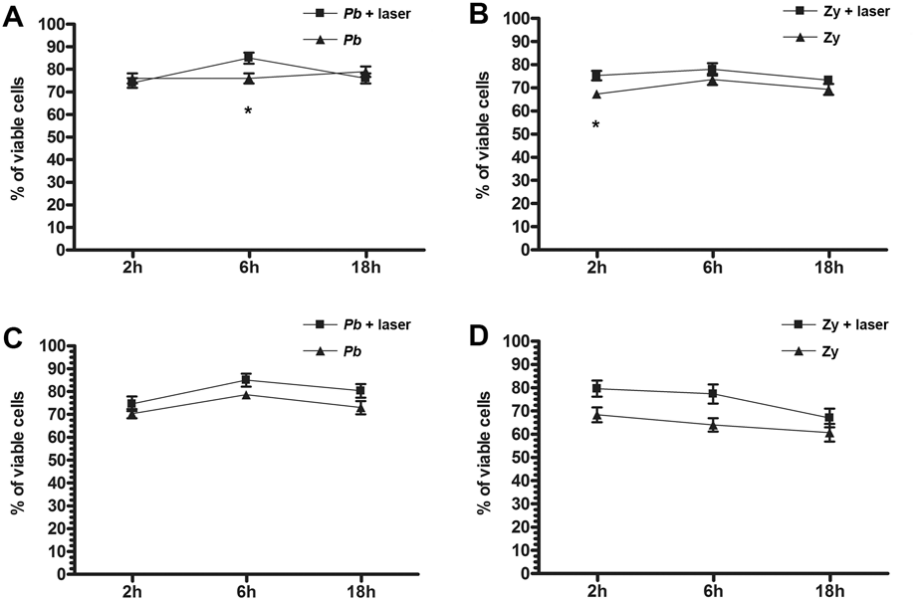
Kinetic behavior of PMN viability. Kinetics of PMN cell viability in irradiated and non-irradiated mice after stimuli with: **A**—*Pb*: higher viability for the irradiated PMN at 6 hours—p = 0.0278 (*); **B**—Zymosan: higher viability for the irradiated PMN at 2 hours—p = 0.0274 (*); **C**—*Pb* stimulated and co-cultivated with *Pb*; **D—**Zymosan stimulated and co-cultivated with *Pb*.

Interestingly, the kinetic study of PMN cell viability showed that LLLT was able to sustain a more viable population of neutrophils for the 18-hour time course both after *Pb* infection (Fig. 3A) at 6 hours (p = 0.0278) and also after Zymosan inoculation (Fig. 3B) at 2 hours (p = 0.0274). There was no statistical significant difference between the viability of the PMN from irradiated or non-irradiated mice after co-cultivation with *Pb* for up to 18 hours, though the viability of the irradiated cells was kept at high levels, similarly to the non-irradiated PMN (Figs. 3C and 3D).

### PMN mitochondrial activity after co-cultivation

**Fig 4:**
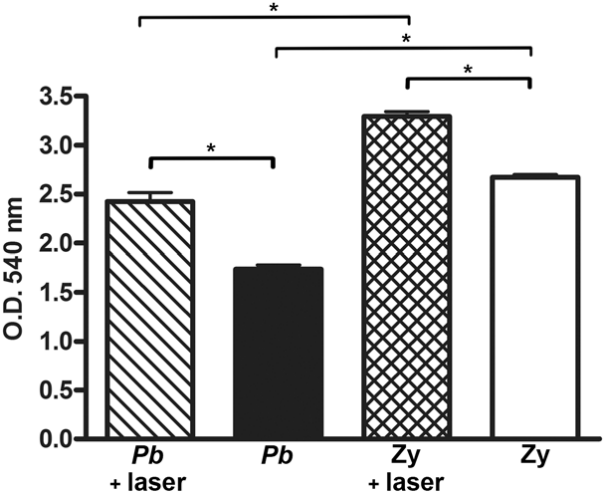
PMN mitochondrial activity after co-cultivation. Mitochondrial activity of light irradiated and non-irradiated PMN recruited by either *Pb* or Zymosan stimuli and co-cultivated with *Pb* in vitro. p = 0.0029 (*) between the irradiated and non-irradiated PMN of the *Pb* group; p = 0.0012 (*) between the irradiated PMN of *Pb* group and irradiated PMN of the Zy group; p = 0.0004 (*) between the irradiated and non-irradiated PMN of the Zy group; p = 0.0001 (*) between the non-irradiated PMN of the *Pb* group and the non-irradiated PMN of the Zy group.

After being co-cultivated with *Pb*18 the PMN recruited by either the *Pb* infection or Zymosan inoculation showed a significantly higher mitochondrial activity (p = 0.0029 and p = 0.0004, respectively) if they had been previously light irradiated *in vivo* (Fig. 4). In addition, the Zymosan irradiated group had a significantly higher mitochondrial activity (p = 0.0012) than the *Pb* irradiated group, while the non-irradiated Zymosan group also had a significantly higher mitochondrial activity (p = 0.0001) than the non-irradiated *Pb* group (Fig. 4).

### PMN protein production

**Fig 5:**
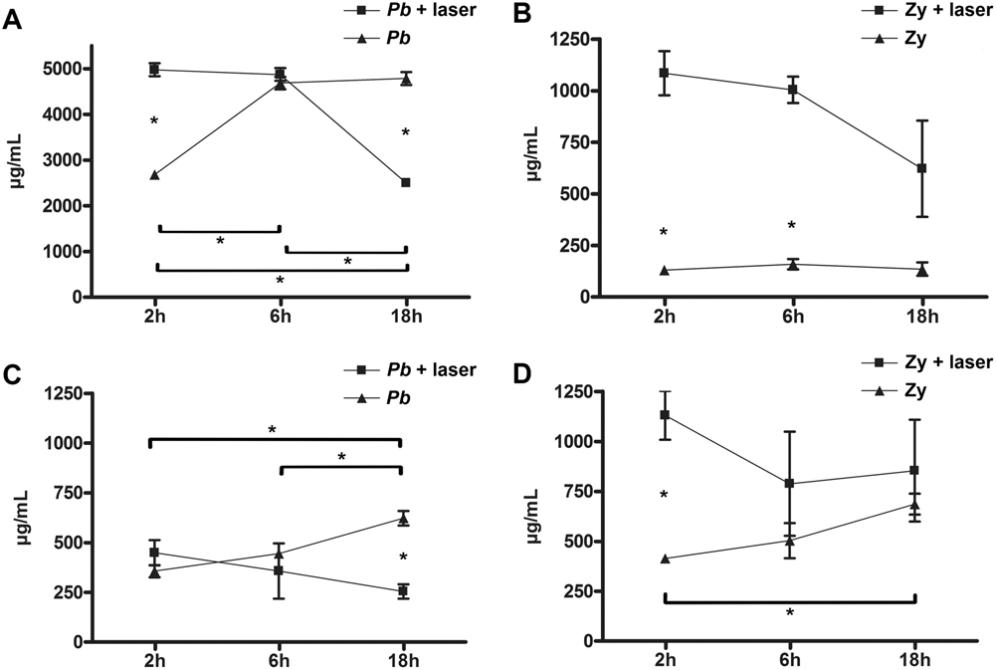
PMN protein production. Protein production kinetics of irradiated and non-irradiated PMN. **A**—*Pb* infected mice (non-irradiated and irradiated); **B**—Zymosan (Zy) inoculated mice (non-irradiated and irradiated); **C—**PMN recruited by *Pb* infection and co-cultivated with *Pb*; and **D—**recruited by Zy inoculation and co-cultivated with *Pb*. **A:** p = 0.002 (*) between 2 and 6 hours of *Pb* non-irradiated; p = 0.0001 (*) between *Pb* non-irradiated and *Pb* irradiated at 2 hours; p = 0.001 (*) between 2 and 18 hours of *Pb* irradiated PMN; p = 0.001 (*) between 6 and 18 hours of *Pb* irradiated PMN; and p = 0.001 (*) between the *Pb* irradiated and non-irradiated PMN at 18 hours; **B**: p = 0.009 (*) between Zy non-irradiated and Zy irradiated at 2 hours; and p = 0.003 (*) between Zy non-irradiated and Z irradiated at 6 hours; **C:** p = 0.005 (*) between the non-irradiated PMN of the *Pb* group at 2 and 18 hours of co-cultivation; p = 0.0481 (*) between the non-irradiated PMN of the *Pb* group at 6 and 18 hours of co-cultivation with *Pb*; and p = 0.002 (*) between the non-irradiated and irradiated PMN at 18 hours of co-cultivation; **D:** p = 0.0043 (*) between the non-irradiated and irradiated PMN at 2 hours of co-cultivation; p = 0.0069 (*) between the non-irradiated PMN at 2 and 18 hours of co-cultivation.

Protein production was significantly enhanced with LLLT at earlier evaluation periods when compared to the non-irradiated groups (p = 0.0001 and p = 0.009 for *Pb* and Zymosan recruited PMN, respectively). The kinetics of protein production illustrates an intriguing crescent behavior for non-irradiated/*Pb* stimulated PMN (p = 0.002) and an opposite decaying curve for the LLLT/*Pb* neutrophils (p = 0.001); this decaying curve was also obtained with the highly activated PMN from the irradiated Z groups (Fig. 5). Likewise, after *Pb* co-cultivation, the kinetic production of proteins by irradiated PMN (*Pb* and Zymosan recruited) underwent decreasing curves that were distinct from the growing curves produced by the non-irradiated groups; this led to very distant values between the non-irradiated and the irradiated groups at 18 hours of co-cultivation (p = 0.002) (Fig. 5). In summary, after 2 hours the *Pb* or Zymosan recruited PMN were significantly more metabolically active than their non-irradiated counterpart (p = 0.0001 and p = 0.009, respectively); in addition, after 18 hours of co-culture the *Pb*-recruited PMN that did not receive LLLT were significantly more active than the *Pb* irradiated group (p = 0.002); the Zymosan-recruited group also showed an initial disparity between irradiated and non-irradiated groups (p = 0.0043) when co-cultivated with the *Pb*; this disparity was neutralized after 18 hours of the co-culture (Fig. 5).

### Quantification of reactive oxygen species produced by PMN

**Fig 6:**
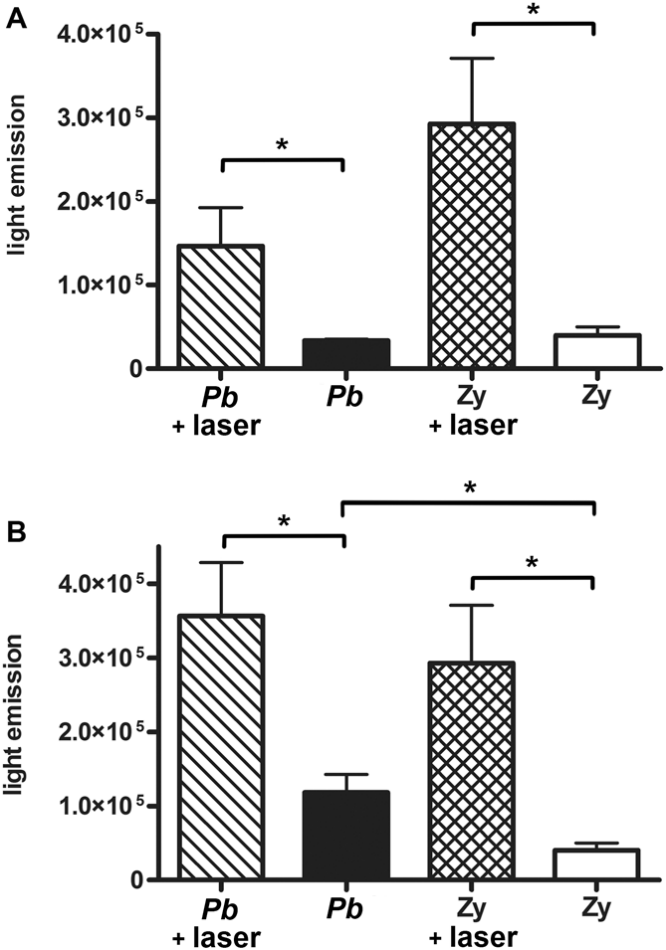
Quantification of reactive oxygen species (ROS) produced by PMN. Luminol chemiluminescence of ROS (photon counts x 10^4^ per second) by light irradiated and non-irradiated PMN recruited by **A—** *Pb* or Zymosan (Zy): p = 0.0425 (*) between non-irradiated and irradiated PMN of *Pb* groups; p = 0.0325 (*) between non-irradiated and irradiated PMN of Zy groups; and **B—** *Pb* or Zy co-cultivated with *Pb*: p = 0.0356 (*) between the irradiated and non-irradiated PMN of the *Pb* group; p = 0.0325 (*) between the irradiated and non-irradiated PMN of the Zy group; p = 0.0406 (*) between the non-irradiated PMN of the *Pb* and Zy groups.

As seen in Fig. 6A, a significantly higher amount of ROS production, as measured by chemiluminescence, was seen with PMN from LLLT treated mice for both *Pb* and Zymosan groups (p = 0.0425 and p = 0.0325, respectively). In the co-cultivated groups, the light irradiated PMN consistently produced a significantly higher amount of ROS than their non-irradiated counterparts (p = 0.0356 and p = 0.0325 for the *Pb* and Zymosan recruited PMN, respectively) (Fig. 6B). The non-irradiated *Pb* PMN also produced more ROS than the Zymosan non-irradiated PMN after co-cultivation (p = 0.0406) (Fig. 6B).

### Fungicidal capacity of PMN

**Fig 7:**
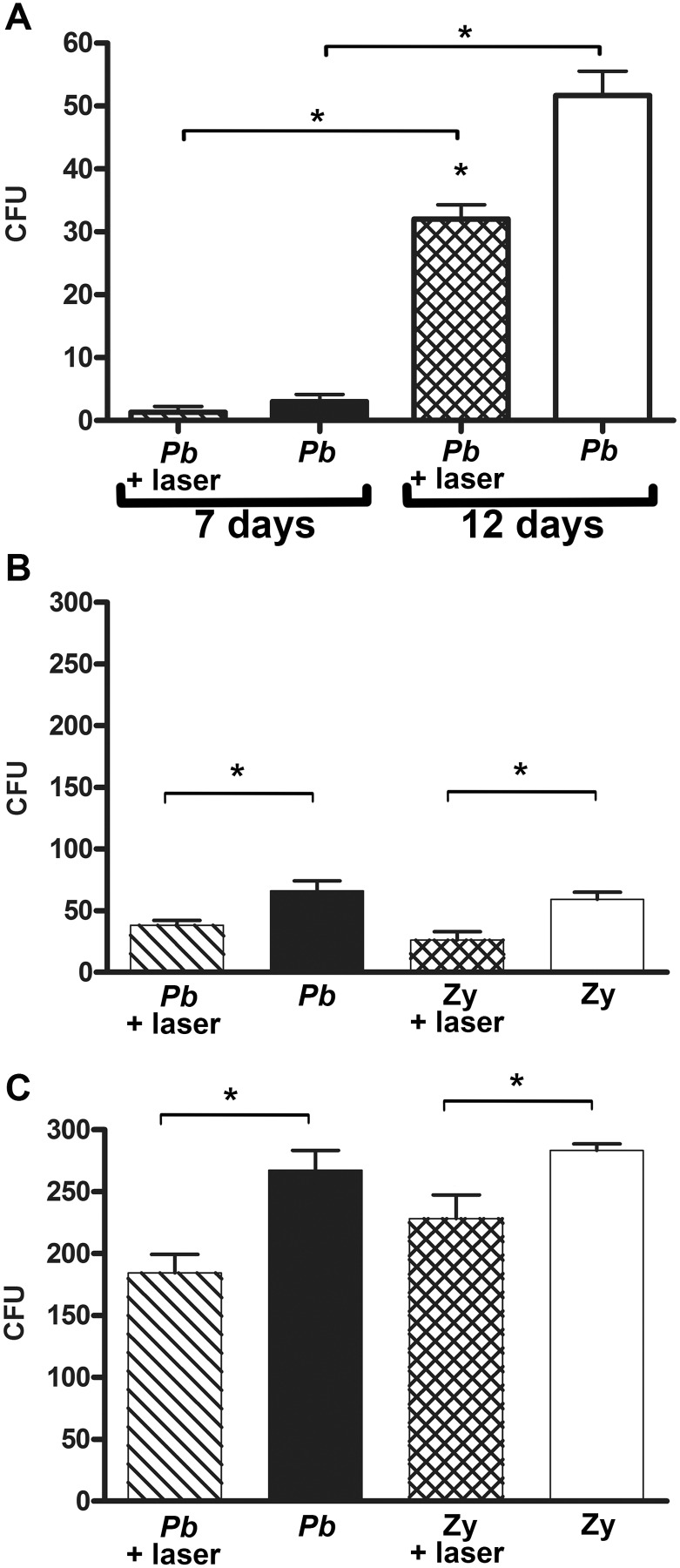
Fungicidal capacity of PMN. **A—**Colony forming units (CFU) of *Pb* at 7 and 12 days evaluation for non-irradiated and irradiated PMN. p = 0.0002 (*) between non-irradiated and irradiated PMN after 12 days of fungal growth; p = 0.0118 (*) between the irradiated groups at 7 and 12 days of fungal growth and p = 0.0003 (*) between the non-irradiated groups at 7 and 12 days of fungal growth. The colony forming units of *Pb* after co-cultivation with PMN recruited by *Pb* infection or Zy inoculation are shown after: **B—**7 days: p = 0.0369 (*) between the irradiated and non-irradiated PMN of the *Pb* group; p = 0.0232 (*) between the irradiated and non-irradiated PMN of the Zy group; or **C—**12 days: p = 0.0193 (*) between the irradiated and non-irradiated PMN of the *Pb* group; p = 0.0492 (*) between the irradiated and non-irradiated PMN of the Zy group.

LLLT treatment of mice was able to induce a higher fungicidal capacity in PMN cells, which was indirectly shown by a significantly lower number of *Pb* colonies growing from material isolated from the air pouches when evaluated after a 12-day growth period (p = 0.0002) (Fig. 7A). Moreover, LLLT was able to induce a significantly higher fungicidal capacity in PMN recruited by either *Pb* or Zymosan after 7 (p = 0.0369 and p = 0.0232, respectively) (Fig. 7B) or even after 12 days (p = 0.0193 and p = 0.0492, respectively) (Fig. 7C) of co-cultivation with *Pb*. Nevertheless, none of the groups was able to totally inhibit the growth of the fungi.

## Discussion

LLLT treatment of the mouse femurs elicited a more active PMN population that could better deal with the *Pb* infection. This was warranted by the characterization of protein levels, mitochondrial activity and ROS assessment that all together showed that PMN from light-irradiated mice were more metabolically active and also produced more ROS, thus being more fungicidal in the actual lesion, and more fungicidal even after a later ex vivo re-exposure to the fungus.

PMN from infected patients may inactivate *Pb* [36] and are considered important as immune attack cells that contribute to the host response against this fungal infection, especially in the early stages of PCM [37]. Nevertheless, neutrophil functions such as fungal killing require activation by cytokines and other elements of the immune system [37]; IFN-γ, TNF-α, granulocyte-macrophage colony-stimulating factor (GM-CSF) and IL-15 are some examples of factors that can activate human neutrophils to carry out increased fungicidal activity by a mechanism dependent on production of ROS such as H_2_O_2_ and superoxide anion [38,39].

The more severe outcome of a *Pb* infection after depletion of PMNs has been previously published [40]; susceptible neutrophil-depleted mice displayed uncontrolled inflammatory responses, while normal resistant mice produced well-balanced Th1/Th2 responses [41] and thus were able to better clear out the fungi [40]. Moreover, normal resistant mice were stronger against the *Pb* infection and had highly activated PMN, in contrast to less activated neutrophils and macrophages seen in susceptible mice [32,42].

Furthermore, defects in PMN activation also correlate with the lack of fungicidal activity; a mutation in the CD40L gene and the lack of CD40L expression by activated T cells are examples of this [2]. An efficient Th1 immune response characterized by sufficient IFN-γ production and the satisfactory activation of phagocytic cells is required to eradicate the *Pb* infection [3]. CD40L-deficient patients showed a T cell response that yielded lower IFN-γ and higher IL-4 and IL-5 production, which led to a higher susceptibility to PCM infection [2].

Since the PMN from healthy human subjects can readily phagocytose the *Pb* cells [43], it has been proposed that neutrophil deficiencies must be present in PCM patients, especially related to the capacity of PMN to phagocytose and destroy this fungus [44–47]. Some previous studies suggested that only neutrophils from pre-sensitized mice could inactivate *Pb in vitro* [45,48]. The concept of activating PMN to produce an improvement in their fungicidal capacity is not completely new. Previously, the enhanced candidacidal activity of PMN and the improved ability of PMN to kill *Blastomyces dermatitidis in vitro* was achieved with an intraperitoneal injection of homologous antigen in *B*. *dermatitidis*-immune mice [27]. Stimulation of sensitized spleen cells with specific antigen can also be helpful to activate PMN for improved microbicidal activity [26].

We aimed to activate the “*Pb*-fighting PMN” with a new method never before utilized for this purpose, namely LLLT delivered to the bone marrow of the femurs of mice. This technique should be completely safe, as it has no known contra-indications and is quick and easy to perform. LLLT is a non-ionizing, non-thermal type of radiation that is known to improve tissue healing [20,21,49], whilst activating several signaling pathways related to cell proliferation, survival, repair and regeneration [19,50–54].

Antifungal drugs may lead to side-effects [16] and may induce development of drug-resistance, which in the case of *Pb* is primarily mediated by increased melanization [18]. However there is no report related to the use of LLLT producing any development of resistance in microorganisms. Moreover, antifungal drugs such as itraconazole have been implicated in producing relapses in a percentage of patients treated for PCM; 50% of these recurrences occurred after 36 months after discontinuing antifungal treatment [17], which is undeniably a considerable interval.

Both the subcutaneous infection with *Pb* and inoculation of Zymosan trigger a marked neutrophil response [55]. Zymosan consists of a mixture of fungal cell wall and intracellular components, amongst which beta-glucans are the most important and elicits many inflammatory responses, such as the production of ROS and cytokines that are involved in phagocytosis of microorganisms by neutrophils and macrophages [56]. Although Zymosan alone did not induce neutrophils as much as the *Pb* itself, LLLT produced bone marrow stimulation that was translated into a higher migration of activated PMN also in the Zymosan group.

The PMN of the Zymosan group, either irradiated or non-irradiated, were always more metabolically active than the *Pb*-exposed cells when co-cultivated with *Pb*. The neutrophils that were facing the *Pb* for the first time showed a higher mitochondrial activity than the cells that were re-exposed to this fungus. Interestingly, Zymosan did provide a more controlled (and thus more beneficial) host immune response than *Pb*, probably due to its recognition by TLR-2 and dectin-1 receptors leading to production of IL-10 [57]. The presence of activated neutrophils even in the Zymosan-irradiated groups showed that LLLT can serve as a tool to activate the bone marrow to produce an improved host defense especially against pathogens that require a rapid attack by the innate immune system. LLLT did not elevate the number of migrated PMN after *Pb* infection, although the neutrophils that were recruited by *Pb* alone were not as effective as the ones that were also light stimulated.

Alpha-1–3 glucan is the major cell-wall component of the yeast phase of *Pb*, therefore, Zymosan and *Pb* share the presence of glucans in common. Glucans are recognized by the c-type lectin, dectin1, as well as by CR3 complement receptor and lactosylceramide [56]. Since we did not add complement to the co-culture, we can surmise that the recognition of *Pb* by the neutrophils during the *in vitro* experiment was by a non-opsonic process. We cannot however, rule out the presence of complement in the exudate at the site of the subcutaneous air pouch. In addition, the air pouch technique was elected over the intraperitoneal [58], intra-oral [59] or intratracheal [60] routes due to its potential to raise a wide pool of almost pure neutrophils and yet localized and controlled *Pb* infection [32]; in that way, LLLT was securely delivered to the bone marrow and not to fungal cells, guaranteeing that the *Pb* killing was in fact due to neutrophil roles and not the laser acting directly upon the fungi.

In wound healing studies, LLLT has been observed to stimulate inflammation in some circumstances [23,24], which would be consonant with our goal of combating infections. Conversely, LLLT is often used to reduce the inflammatory response, and dampen down pro-inflammatory signaling [19,61–63], which was clearly not our focus with the present study. In fact, studies utilizing different models of acute inflammation have presented a declined edema formation and diminished neutrophil influx after LLLT [64–66].

According to these aforementioned studies, we showed that the quantity of the recruited neutrophils was diminished with LLLT, which could have been interpreted as an anti-inflammatory response; however, the level of activation of these cells was significantly improved with the use of LLLT, which was indeed applied to the bone marrow and not to the actual air-pouch. Interestingly, it has been postulated that LLLT may be potentially pro-inflammatory in the absence of antioxidants, while it can act as an anti-inflammatory stimulus when in the presence of sufficient antioxidants [67]. Moreover, neutrophils from patients with PCM are functionally deficient against suspensions of live *Pb* [44,45,68]; these neutrophils degenerate during the process of phagocytosis [4]. Thus, even though the neutrophil influx was higher in the non-irradiated group, this infiltrate was less efficient than the light-stimulated PMN.

The stimulation of bone marrow by LLLT seems to require an additional stimulus by the fungal cells, since the animals inoculated only with saline solution did not show increased recruitment of neutrophils after laser stimulation. Thus, the LLLT mechanism in our study could be described as biomodulatory [67] rather than pro-inflammatory, as if the PMN were primed to respond better against the invasion by fungal cells. LLLT typically leads to an increase in mitochondrial activity [69], and consequent induction of the cell-cycle with the synthesis or release of growth factors, interleukins, cytokines etc [70].

The higher mitochondrial activity seen in the LLLT group could be correlated with the protein production of these cells, while the kinetics of protein production was different among the irradiated and non-irradiated groups. Two hours after PMN extraction the neutrophils of the LLLT group were highly activated and showed a tendency to decrease until they were poorly activated after 18 hours. By contrast, the non-irradiated PMN started as less activated and began to produce higher quantities of proteins as time passed by. This same shaped curves (decreasing for illuminated PMN and increasing for non-illuminated PMN) were obtained for both *Pb* and Zymosan treated groups after they were extracted from the air-pouches and even after they were placed in contact with *Pb in vitro*.

The neutrophils from the mice that did not receive LLLT only achieved the same level of initial activation of the LLLT group after they were cultivated for 18 hours; or co-cultivated for 6 hours along with *Pb*. For the Zymosan group, not even after 18 hours of culture or co-cultivation did the non-illuminated neutrophils achieve the same degree of protein production as the LLLT-group cells. Even the cells that were facing *Pb* for the first time (Zymosan group co-cultivated with *Pb*) were more capable of dealing with this pathogenic fungus once they had been activated by the light.

The appropriate activation of phagocytic cells and particularly the production of ROS by nicotinamide adenine dinucleotide phosphate oxidase are important for the control of fungal infections [2,14]. Our present results show that *Pb* is able to activate the oxidative burst of neutrophils and that these cells are efficient in killing *Pb*, confirming earlier data from our group that showed that PMNs from resistant mice are more efficient in killing *Pb* than PMNs from susceptible mice [32]. In addition, the outbred Swiss mice utilized here were shown to be resistant since their survival rate after *Pb* infection was similar to that of A/J or A/Sn resistant mouse strains [71]; accordingly, LLLT stimulated even further the “already more efficient” [32] PMN from resistant mice.

The PMN from light-irradiated mice produced more ROS than their respective control groups, whether they were recruited through *Pb* or Zymosan inoculation. Rodrigues et al. [38] activated normal human neutrophils *in vitro* by using cytokines (IFN-γ, TNF-α, GM-CSF), thereby increasing their fungicidal activity against *Pb*, and showed the participation of ROS in this process. The same group also showed the suppressive effect of IL-10 in the same process [3].

We could also establish a good correlation between the ROS production and the fungicidal activity of PMN; CFU counting demonstrated that the material from irradiated mice had less viable *Pb* cells, after 12 days growth on solid media. Furthermore, PMN from light-irradiated mice that were re-exposed to *Pb* retained their higher fungicidal activity. Moreover, even the PMN from the LLLT-Zymosan group that underwent an initial contact with *Pb in vitro* were able to substantially impair the growth of *Pb*.

In the literature concerning co-cultivation studies between PMN and *Pb*, there is a report of a fungistatic (not a fungicidal effect), and only after a long incubation time with *Pb* (72 hours). Although PMN treated with IFN-γ did show better killing abilities (not against all studied strains), tumor necrosis factor-α and IL-8 did not improve PMN antifungal capacity [47]. In our study LLLT appeared to be an effective approach since it did enhance the fungicidal capacity of PMN after co-cultivation. It should be noted that LLLT was delivered to the mouse femur to activate the bone marrow, not to the actual PMN *in vitro*. The effect of the LLLT enabled the recruited PMN to fight the highly virulent *Pb*18 strain [28] both *in vivo* and in co-culture; nevertheless, the subcutaneous air pouch route utilized herein does not represent the natural course of PCM within the patients (inhaled fungal cells), so the results of this study may not be overestimated.

### Conclusions

PMN activation through LLLT to the bone marrow led to a higher cell activity that correlated with two main effects: enhancement of innate immunity, shown by the higher yield of ROS and inhibition of *Pb* CFU in the lesion; and possible stimulation of acquired immune response shown by the increased yield of proteins seen in the LLLT groups. Finally, it is worth mentioning that although LLLT could be an effective and totally safe technique to activate fungicidal neutrophils, it was still not enough to eradicate the PCM; as previously stated, the phagocytic activity of PMN is considered not sufficient to entirely kill *Pb* [4]. Further study is warranted to see if different LLLT parameters, different sites of mouse irradiation or even distinct *Pb* infection routes could produce even better results from this promising technique.

## Supporting Information

S1 Fig Subcutaneous air-pouches of the Saline groups. Clinical appearance of the air-pouches on the dorsum after skin flap procedure in mice inoculated with: **A—**saline; and **B—**saline followed by LLLT. Both groups reveal no neutrophil influx.
(TIF)
