# Supplementary figures and images for: Low-level Laser Therapy to the Mouse Femur Enhances the Fungicidal Response of Neutrophils against Paracoccidioides brasiliensis
Source: PLoS Negl Trop Dis. 2015 Feb 12;9(2):e0003541. doi: 10.1371/journal.pntd.0003541 (PMC4326423; doi:10.1371/journal.pntd.0003541)

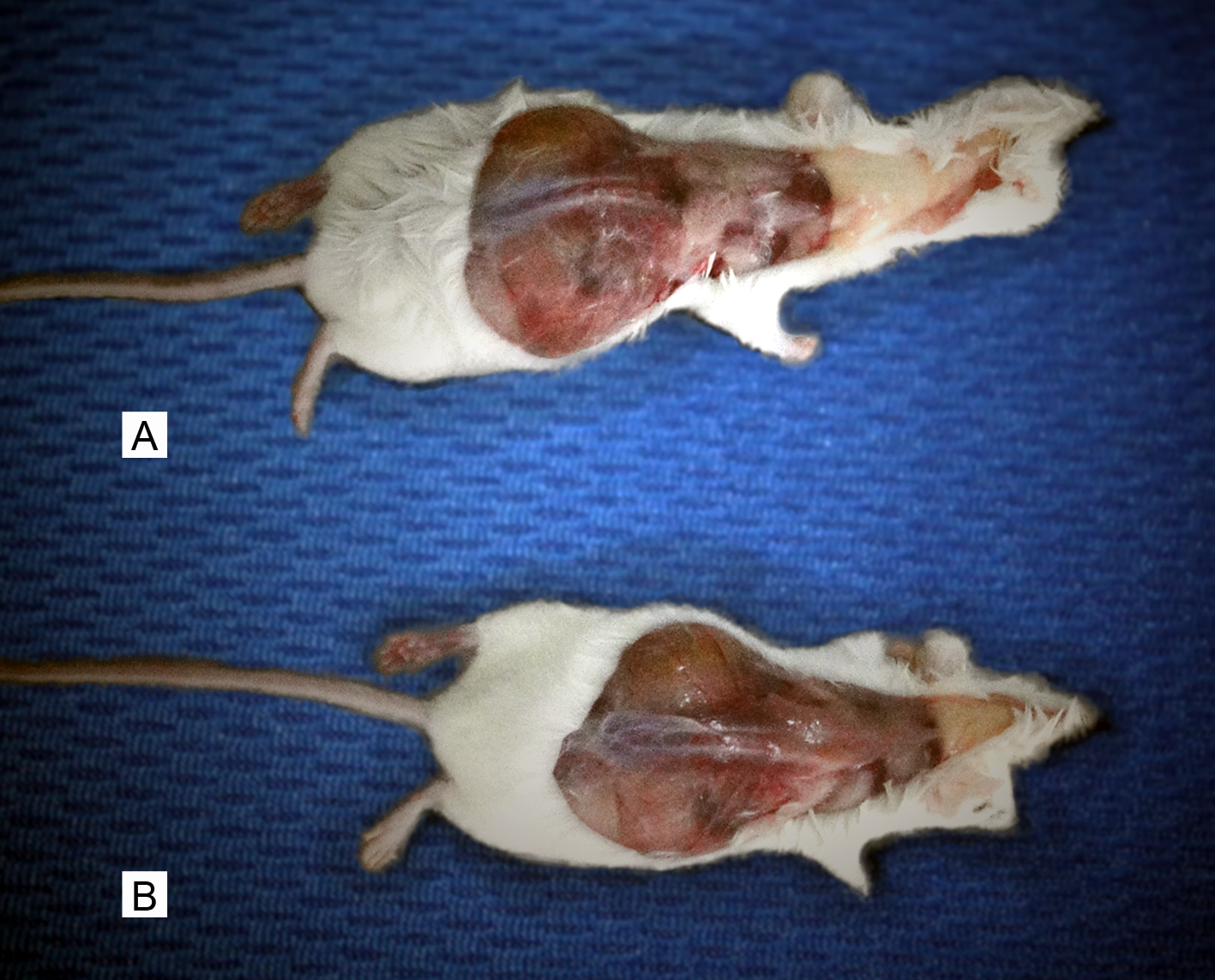

Supplement: S1 Fig — Clinical appearance of the air-pouches on the dorsum after skin flap procedure in mice inoculated with: A—saline; and B—saline followed by LLLT. Both groups reveal no neutrophil influx. (TIF) [file pntd.0003541.s001.tif]
